# Giant Basal Cell Carcinoma Within a Marjolin's Ulcer: A Case Discussion

**DOI:** 10.7759/cureus.67629

**Published:** 2024-08-23

**Authors:** Steven Latta, Silas J Helbig, Karine Kasti, Elizabeth Paulus

**Affiliations:** 1 College of Medicine, Florida International University, Herbert Wertheim College of Medicine, Miami, USA; 2 Surgical Oncology, University of Miami, Miami, USA

**Keywords:** marjolin's ulcer, dermatology, basal cell carcinoma (bcc), squamous cell carcinoma (scc), hiv

## Abstract

Marjolin's ulcers are cutaneous malignancies that arise from chronic wounds, often secondary to burns. While squamous cell carcinoma is the most prevalent type, rare instances of other tumors, such as basal cell carcinoma, have occurred. These tumors are challenging to treat due to their high recurrence rate and aggressive behavior. In this report, we present the case of a 76-year-old male with a history significant for polysubstance use, human immunodeficiency virus (HIV), hepatitis C, and Agent Orange exposure who presented with a large fungating basal cell carcinoma secondary to a non-healing wound. Additionally, several other cutaneous malignancies were present, including a large verruciform squamous cell carcinoma adjacent to a Marjolin's ulcer. These lesions were managed with surgical excision, followed by radiation therapy due to suboptimal margins.

## Introduction

Marjolin's ulcers (MUs) are an uncommon form of cutaneous malignancies that arise from chronic inflammatory changes within a wound. While burns are the most common culprit, any non-healing wound has the potential to undergo malignant transformation. The incidence of MUs varies, but it is estimated that 1.7% of chronic wounds will result in malignant transformation [1]. The vast majority of these ulcers form cutaneous squamous cell carcinomas (SCCs), but in rare instances, the formation of basal cell carcinoma (BCC) or malignant melanoma has occurred. Regardless of their subtype, delay in treatment may be detrimental to patient outcomes as MUs are frequently more aggressive and more susceptible to early metastasis [1,2].

## Case presentation

**Figure 1:**
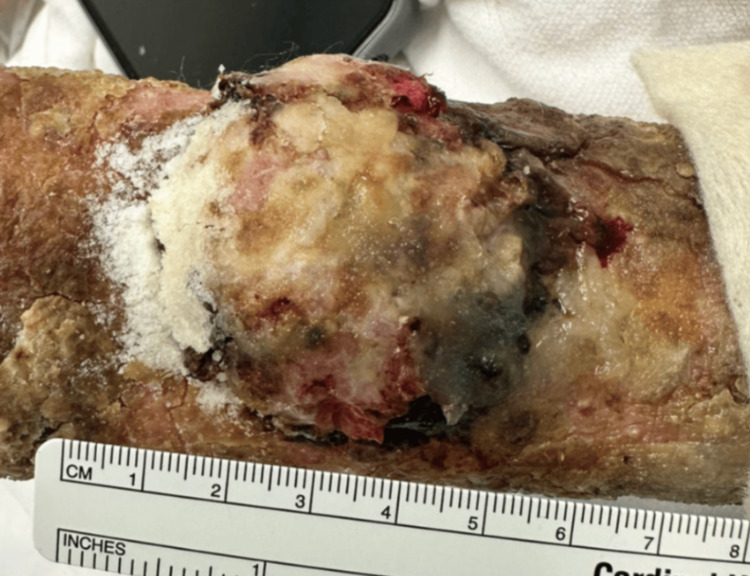
Distal lesion on the left extremity presenting as a fungiform mass.

**Figure 2:**
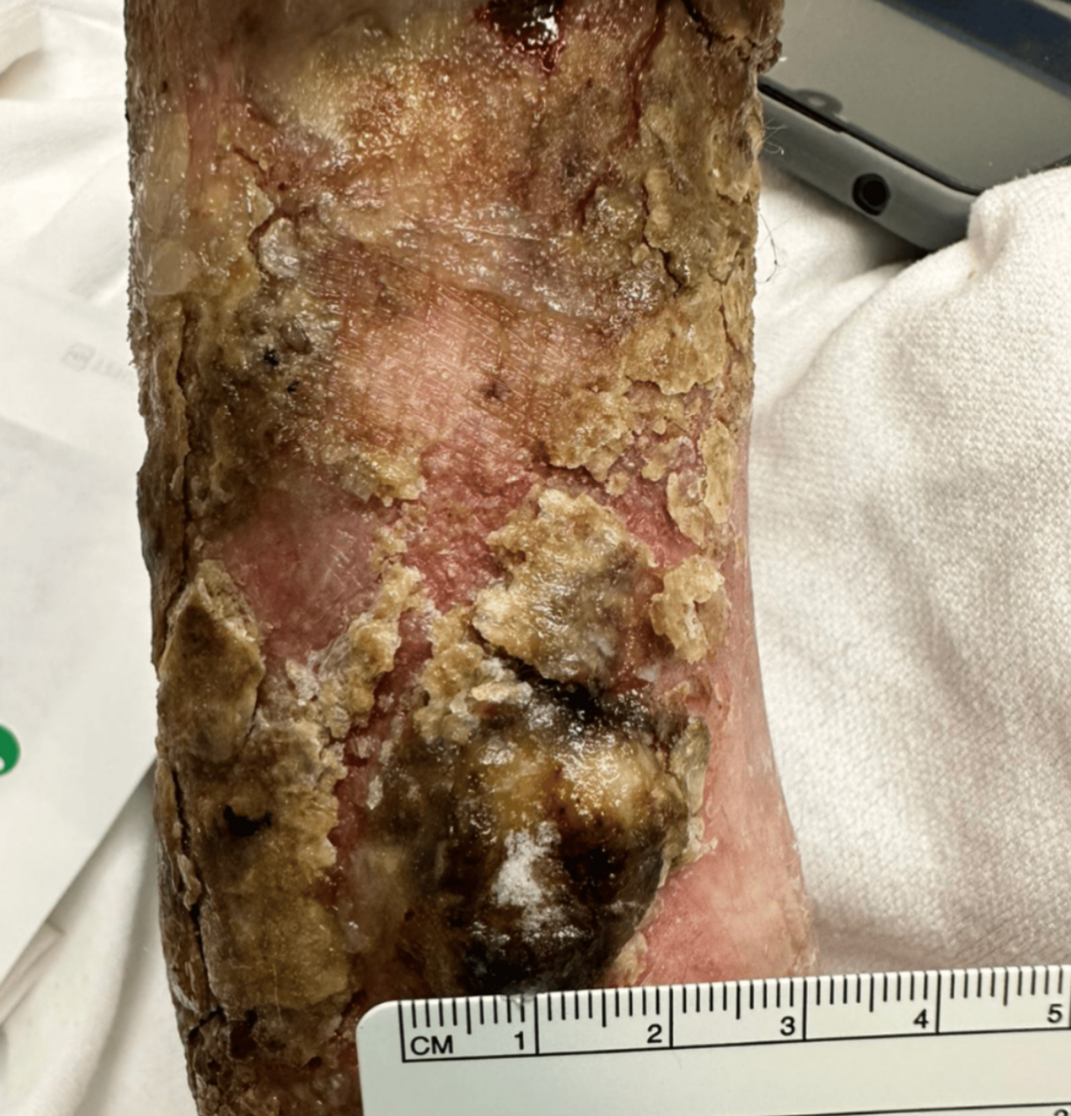
Proximal lesion on the left extremity with crusting, scale, and poorly defined borders.

A 76-year-old male patient presented to an academic medical center due to a large palpable mass on his left distal forearm. He first noticed the lesion a few years prior after cutting his arm from a fall. He reports using over-the-counter antibiotic ointment to the wound daily over the next few months, but despite treatment, the wound failed to heal. Upon presentation, the mass was non-painful but would occasionally itch and bleed. Physical examination of his left arm revealed a large fungating mass on the distal aspect of the dorsal forearm (Figure 1). Proximal to that lesion, a smaller mass with poorly defined borders, crust, and scale was also noted (Figure 2). The patient additionally had numerous solar lentigos and actinic keratosis present on his shoulders, back, and scalp. 

Upon review of the patient's history, he is both an American-born citizen and a retired veteran. He served in the Vietnam War and reports extensive exposure to Agent Orange during service. From the war, he developed post-traumatic stress disorder and later turned to polysubstance use to cope with his experiences. As a consequence of past intravenous drug use, he acquired both human immunodeficiency virus (HIV) and hepatitis C over 20 years ago. He is compliant with both his ledipasvir/sofosbuvir therapy and highly active antiretroviral therapy (HAART), as reflected by his last CD4 count of 615 taken one year ago, a significant increase from his previous low of 358. The patient reports being treated for multiple smaller cutaneous malignancies in the past.

**Figure 3:**
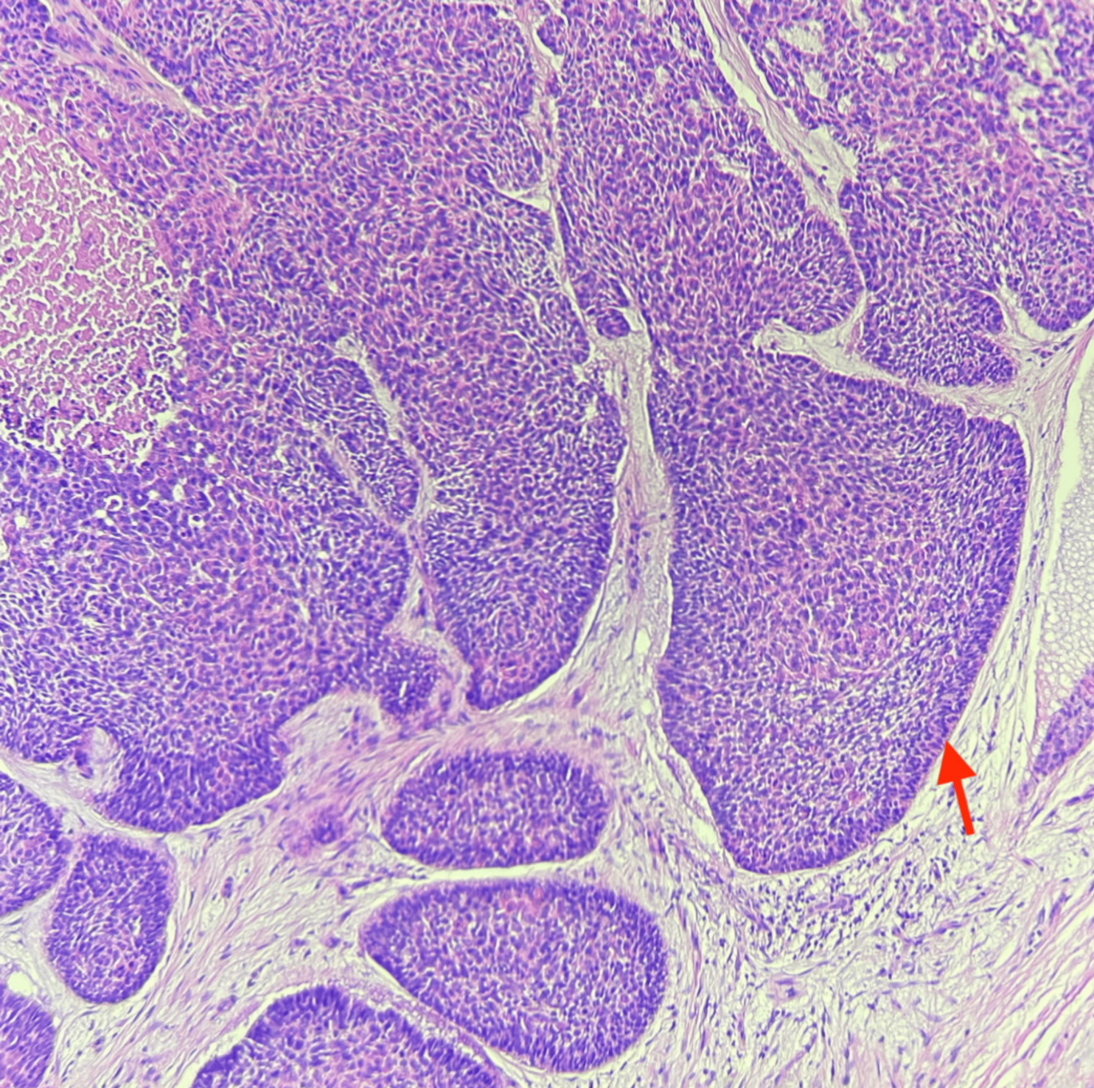
Histology showing nests of basophilic cells with scant cytoplasm, hyperchromatic nuclei, and the classic peripheral palisading of nuclei seen in BCC (red arrow). BCC: basal cell carcinoma

A punch biopsy of the two lesions was performed and histopathology confirmed both lesions were malignant. The distal lesion was determined to be a nodular ulcerated BCC (Figure 3), and the proximal lesion was a verrucous SCC. 

**Figure 4:**
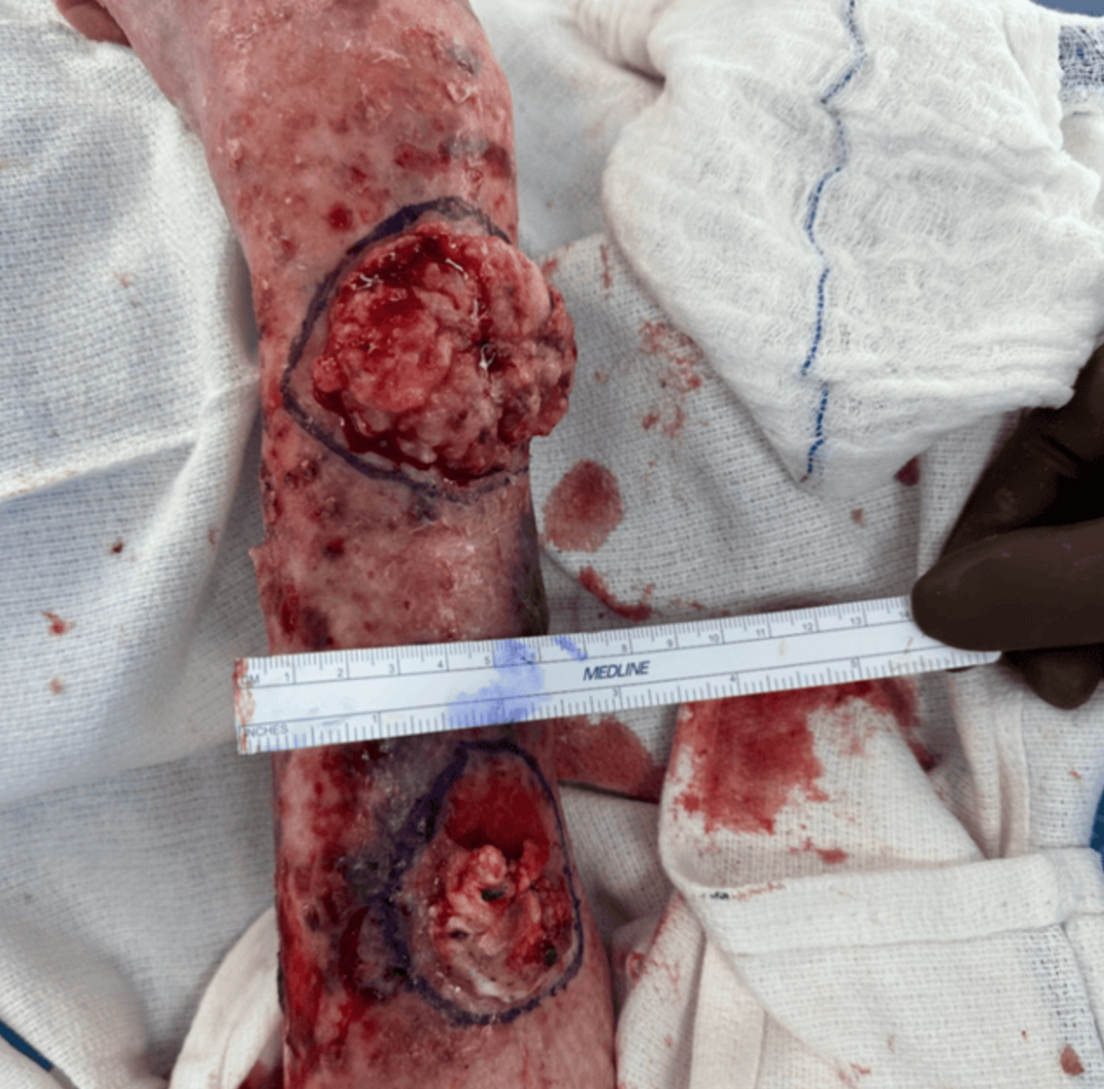
Gross appearance of the BCC (distal) and SCC (proximal) after surgical debridement. BCC: basal cell carcinoma; SCC: squamous cell carcinoma

**Figure 5:**
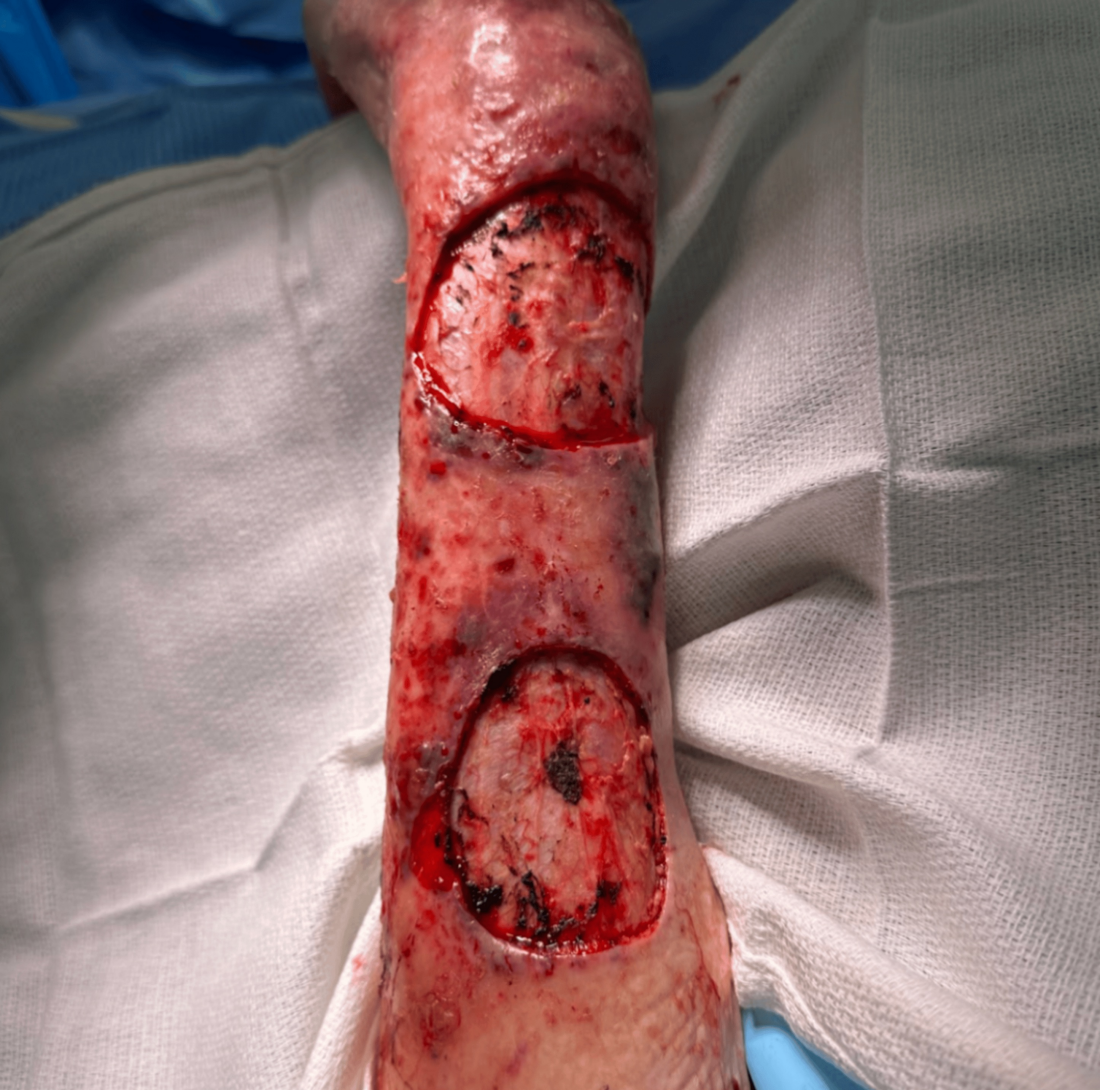
Appearance of the lesions after excision past the dermis.

The patient elected to undergo surgical removal of the tumors under the care of a multidisciplinary team, including surgical oncology, anesthesiology, and plastic surgery. The masses were first marked, cleaned, and debrided to expose the tumors fully (Figure 4). The tumors were then surgically excised past the dermal layer, with margins based on gross examination (Figure 5). Following excision, a wound vacuum sealant was used to cover the lesions with the plan to undergo skin grafting at a later date.

The dimensions of the SCC were 4×4×1.2 cm, and the surgical margins were histologically confirmed to be free of tumor. The BCC was larger and measured 6.3×5×1.5 cm. While all of the margins were free of tumor, the BCC was <1 mm away from the deep margin. These findings were discussed with the patient who elected to pursue additional radiation treatment in the outpatient setting. 

## Discussion

MU is an aggressive cutaneous malignancy that arises in chronic non-healing wounds. The natural history of MUs includes the initial insult, which then undergoes either an acute or a chronic phase. Chronic MUs involve a period of time, known as the latency period, between the initial injury and the formation of an MU. While the average latency period varies widely, it is typically greater than a decade. In contrast, acute MUs have much shorter latency periods and will present within 12 months of cutaneous injury [2-4]. The most common histology for MUs is by far SCC; however, other tumors including BCC and melanoma have been described. Kanth et al. conducted a systematic review containing 1,016 patients with MUs and reported that approximately 94% were SCC, 2% were BCC, and 4% had other tumors [3]. Bazaliński et al. reported SCC presents more commonly in chronic MUs, while BCC typically occurs in acute MUs [5].

Despite MUs being well known, controversy remains in the management of MUs as they frequently recur after treatment [3]. For most treatment guidelines, providers often turn to the recommendations provided by the National Comprehensive Cancer Network (NCCN); yet the NCCN does not have any specific guidelines for the workup and management of MU-associated malignancies [6]. Surgery is the most common treatment for MUs, with local excision being the most common procedure. In more advanced cases, amputation may be necessary [7]. The utility of lymphadenectomy and sentinel lymph node biopsy in these patients was variably reported, with conflicting evidence on the efficacy. Radiation has been shown to have efficacy in treating MUs that are difficult to resect or have inadequate margins [3]. 

In this case, the patient's wound expanded within months of the initial injury, suggesting an acute MU. This observation is consistent with Bazaliński et al.'s claim that the majority of acute MUs present as BCCs [5]. This patient presented with a BCC measuring 6.3 cm in diameter. Conventionally, a BCC is deemed giant once it surpasses a diameter of 5 cm [8]. Such occurrences of a giant BCC in an MU are exceptionally rare and, to our knowledge, have not been previously reported. The cause for the extreme growth in this patient remains unclear; however, this patient presents several risk factors for malignancy that warrant exploration and discussion.

It is well known that people living with HIV infection have an increased lifetime risk for cutaneous malignancy. HIV patients were shown to have a 5.4-fold higher risk of developing cutaneous SCC and a 1.79-fold higher risk of developing BCC in comparison to the general population [9]. These patients are also more likely to experience SCC and BCC, which are more aggressive in nature and are more often found on the trunk and extremities, versus the head and neck region in immunocompetent individuals [10]. Interestingly, there is no strong evidence correlating the extent of CD4 count decline or HIV viral load to the incidence of skin cancers [10,11]. However, patients with immunocompromising conditions, including those with HIV/acquired immunodeficiency syndrome (AIDS), are at a higher risk of developing chronic non-healing wounds. These wounds may create a favorable environment for the development of MUs [1,12].

Another possible risk factor in this patient is his exposure to Agent Orange. Agent Orange was a tactical herbicide used by the United States during the Vietnam War to defoliate thick jungles and target food crops. Agent Orange has multiple active ingredients, but most notable is the contaminant dioxin 2,3,7,8-tetrachlorodibenzo-p-dioxin (TCDD), which has been identified as one of the more toxic ingredients. Agent Orange exposure has been linked to a number of malignant and non-malignant health conditions. Examples of cancers recognized by the US government as linked to exposure to Agent Orange include bladder cancer, chronic B-cell leukemias, Hodgkin's and non-Hodgkin's lymphoma, multiple myeloma, prostate cancer, lung cancer, and soft tissue sarcomas [13]. Smaller studies have reported an increased incidence of non-melanoma skin cancers in those exposed to Agent Orange compared to the general population [14]. However, evidence is still limited as demonstrated by the latest update in 2018 from the committee on Veterans and Agent Orange through the National Academies of Sciences, Engineering, and Medicine, which concluded there is inadequate evidence to determine whether there is an association between exposure and BCC or SCC [13].

## Conclusions

This case sheds light on the complexity surrounding the management of aggressive cutaneous malignancies, particularly within the context of MUs. It also highlights the complex interplay of various risk factors seen in this patient which could be contributing to the development of such an aggressive cutaneous malignancy. A paucity of information exists regarding the presentation and management of giant BCCs within the context of MUs, both acute and chronic. Specifically, there is a lack of research into the prevalence, epidemiology, risk factors, treatment modalities, and recurrence patterns for giant BCCs within MUs. All of this contributes to the lack of specific guidelines for the management of MUs and emphasizes the necessity for standardized approaches to optimize patient outcomes and reduce the likelihood of recurrence. Clinicians should report cases similar to this so more can be learned about the frequency and characteristics of patients affected.
